# Sleep-disordered breathing-related symptoms and risk of stroke: cohort study and Mendelian randomization analysis

**DOI:** 10.1007/s00415-021-10824-y

**Published:** 2021-10-01

**Authors:** Olga E. Titova, Shuai Yuan, John A. Baron, Eva Lindberg, Karl Michaëlsson, Susanna C. Larsson

**Affiliations:** 1 Unit of Medical Epidemiology, Department of Surgical Sciences, The EpiHub, Uppsala University, Dag Hammarskjölds väg 14 B, 75185 Uppsala, Sweden; 2 Unit of Cardiovascular and Nutritional Epidemiology, Institute of Environmental Medicine, Karolinska Institutet, Stockholm, Sweden; 3 Department of Medicine, University of North Carolina School of Medicine, Chapel Hill, NC USA; 4 Department of Epidemiology, Gillings School of Global Public Health, University of North Carolina, Chapel Hill, NC USA; 5 Department of Medical Sciences, Respiratory, Allergy and Sleep Research, Uppsala University, Uppsala, Sweden

**Keywords:** Stroke, Sleep-disordered breathing, Cohort, Mendelian randomization, Single-nucleotide polymorphisms

## Abstract

**Background:**

Sleep-disordered breathing (SDB) may contribute to development of stroke. However, findings are inconclusive. We investigated whether SDB-related symptoms are associated with incidence of stroke and its types in a general community sample of adult men and women as well as to perform Mendelian randomization (MR) analysis.

**Methods:**

We used data from a cohort of 41,742 Swedish adults (56–94 years of age) who completed questionnaires regarding snoring, cessation of breathing, lifestyle and health characteristics. Participants were followed up for incident stroke and death over 8 years through linkage to the Swedish Registers. Hazard ratios, adjusted for potential confounders, were estimated by Cox proportional hazards regression. MR analyses were performed using single-nucleotide polymorphisms associated with sleep apnea at the genome-wide significance level and summary-level data for stroke and its subtypes from consortia and a meta-analysis of Genome-Wide Association Studies.

**Results:**

In the cohort study, symptoms of disturbing snoring and/or cessation of breathing were associated with increased risk of total stroke (hazard ratio 1.12, 95% confidence interval 1.02–1.24) and intracerebral hemorrhage (hazard ratio 1.59, 95% confidence interval 1.23–2.05) but not with ischemic stroke or subarachnoid hemorrhage. MR analyses showed no association of genetic liability to sleep apnea with the risk of overall stroke or any specific types of stroke or ischemic stroke subtypes.

**Conclusions:**

SDB-related symptoms were associated with increased risk of total stroke, specifically intracerebral hemorrhage, in the observational analyses but not in the MR analyses. There was limited evidence of an association of SDB with ischemic stroke and subarachnoid hemorrhage.

**Supplementary Information:**

The online version contains supplementary material available at 10.1007/s00415-021-10824-y.

## Introduction

Stroke is one the leading causes of long-term disability and mortality worldwide. Hypertension, diabetes, and smoking are among the most commonly described modifiable risk factor for stroke [6]. In addition, evidence suggests that sleep-disordered breathing (SDB) may also contribute to the development of stroke [16–18].

Obstructive sleep apnea (OSA), the most prevalent type of SDB, is characterized by repeated episodes of either partial or full cessation of breathing during sleep. The prevalence of OSA increases with age, and men and obese individuals are more likely to suffer from this condition. Loud snoring, sleep-related breathing problems, and daytime sleepiness are frequent symptoms of OSA [22]. Although SDB is frequently emphasized to be associated with long-term health consequences, this disorder both remains often undiagnosed [19] and admittedly, without often firm causal evidence of disease association. Sleep laboratory studies have demonstrated high prevalence of OSA in stroke patients [12]. However, large prospective cohort studies are needed to investigate whether SDB may increase risk of specific stroke independently of obesity in participants with no history of stroke at baseline.

Previous cohort studies of the association between SDB and subsequent stroke has focused on overall or ischemic stroke and results are inconsistent. Some cohort studies have demonstrated an approximately 2- to fourfold increased risk of incident stroke among participants with OSA [16–18], whereas other studies found no association [3, 32]. No prospective study today has investigated the association between SDB-related symptoms and incidence of hemorrhagic stroke and subarachnoid hemorrhage (SAH). Conventional observational studies are prone to confounding and reverse causality biases that can be reduced by the Mendelian randomization (MR) design [10]. MR is an epidemiologic technique that utilizes genetic variants that strongly associate with the modifiable risk factors to estimate their causal role for the risk of disease [23].

The aim of this study was to investigate the association of self-reported SDB-related symptoms with overall stroke and specific stroke types in a cohort of 41,742 middle-aged and elderly men and women. In addition, we performed a 2-sample MR study to explore whether sleep apnea is associated with overall stroke or any stroke subtype.

## Methods

### Study population

In the primary analysis, we used data from the National Research Infrastructure SIMPLER (Swedish Infrastructure for Medical Population-based Life-course Environmental Research). A detailed description of the cohort infrastructure can be found elsewhere (https://www.simpler4health.se). Information on lifestyle and other risk factors for chronic diseases was obtained via structured questionnaires in 2008–2009 and used as baseline in the present study. From 47,812 participants who completed the questionnaires in 2008–2009, we excluded individuals who died prior to July 1, 2009 (*n* = 44); and those who had missing information on SDB (*n* = 4144). We further excluded 1882 participants with a diagnosis of any stroke before start of a follow-up, as ascertained through linkage to the Swedish National Patient Register. After exclusions, 41,742 participants (18,692 women and 23,050 men) with a mean baseline age of 69 (56–94) remained for analysis. The cohort study was approved by the Swedish Ethical Review Authority and written informed consent has been obtained from the participants.

### Exposure assessment

In 2008/2009, participants completed questionnaires which included information about snoring, cessation of breathing, educational attainment, smoking status, alcohol consumption, weight, height, physical activity, and history of diabetes, hypertension and hypercholesterolemia. Participants reported how often they experienced sleep apnea or cessation of breathing as well as disturbing snoring during the past 3 months with the following options: never; seldom; often; mostly and always. Participants who indicated that they experienced sleep apnea/cessation of breathing or snoring often, mostly or always were defined as having SDB symptoms.

### Case ascertainment and follow-up

Cases of stroke and death were determined through linkage with the Swedish National Patient Register (covering both in- and out-patients) and the Cause of Death Register using the unique personal identity number assigned to each Swedish resident and classified according to the International Classification of Diseases, ICD, 10th Revision codes. The endpoints in the present study were acute ischemic stroke (I63), intracerebral hemorrhage, ICH (I61), subarachnoid hemorrhage (I60), and unspecified stroke (I62). Participants were followed up from July 1, 2009 to the date of diagnosis of CVD, death from any cause, or December 31, 2017, whichever occurred first.

### Two-sample MR analysis

We used publicly available summarized data for genetic associations with overall and ischemic stroke from the MEGASTROKE consortium [15], ICH from the International Stroke Genetics consortium [29] and subarachnoid hemorrhage from a meta-analysis of Genome-Wide Association Studies (GWASs) on intracranial aneurysms [4]. Studies included in the consortia were approved by local research ethics committees and institutional review boards and all participants provided written informed consent. The MEGASTROKE consortium included 446,696 individuals of European ancestry (406,111 non-cases and 40,585 cases of any stroke); the number of cases of ischemic stroke was 34,217 overall, 3373 for large-artery atherosclerotic stroke (LAS), 5386 for small vessel stroke, and 7193 for cardioembolic stroke. The Trial of ORG 10172 in Acute Stroke Treatment criteria was used to subtype ischemic stroke. Summary statistics data for hemorrhagic stroke were available from two meta-analyses of GWASs (ICH: 3223 cases and 3725 non-cases and subarachnoid hemorrhage: 7495 cases and 71,934 non-cases). The MR analysis was approved by the Swedish Ethical Review Authority.

### Instrumental variable selection

Thirty-nine single-nucleotide polymorphisms (SNPs) associated with sleep apnea at the level of genome-wide significance (*p* < 5 × 10^−8^) were identified from a meta-analysis of five cohorts and a previous GWAS including a total of 510,484 participants of European ancestry [8]. These SNPs were replicated in 23andMe and this GWAS analysis was adjusted for sex, age, BMI, genetic principal components and genotype platform [8]. Linkage disequilibrium across 39 SNPs were assessed using the TwoSampleMR package. We used 35 independent SNPs (*r*^2^ < 0.01 and clump distance > 10 kb in European populations) as instrument variables for sleep apnea. The used SNPs for sleep apnea explained around 0.98% of phenotypic variance. The summary-level effect size (beta and standard error) of sleep apnea-associated SNPs were estimated based on the population from 23andMe. Details of the SNPs used as instrumental variables are available in *Supplementary Table I.*

### Statistical analysis

In our analysis based on the SIMPLER’s cohort data, Cox proportional hazards regression models were used to estimate hazard ratios (HR) with corresponding 95% confidence intervals (CI) with age as the time scale and adjusted for sex (as a stratification variable) in the basic model. In a first multivariable model, we additionally adjusted for body mass index (weight divided by the square of height; < 22.5; 22.5–24.9; 25.0–29.9; or ≥ 30 kg/m^2^). In a second multivariable model, we further adjusted for education (less than high school, high school, or university), smoking status (never, former, current smokers), alcohol intake (never drinkers; past or current drinkers of < 1 drink/week; 1– < 7 drinks/week; 7– < 15 drinks/week; 15–21 drinks/week; > 21 drinks/week), walking/bicycling (never/seldom; < 20 min/day; 20–40 min/day; > 40 min/day), exercise (almost never; < 1 h/week; 1 h/week; 2–3 h/week; 4–5 h/week; ≥ 5 h/week), and history of diabetes (yes/no), hypertension (yes/no), and hypercholesterolemia (yes/no). In a sensitivity analyses, we additionally adjusted for cohabitation status (cohabiting *vs* not cohabiting). In the primary analysis, SDB was treated as a dichotomous variable (no SDB symptoms *vs* at least one SDB symptom which occurred often, mostly or always). In secondary analyses, we considered the number of SDB symptoms as an exposure variable: no SDB symptoms, snoring or sleep apnea/cessation of breathing or both snoring and sleep apnea/cessation of breathing.

Schoenfeld residuals were used to test the proportional hazards assumption. No interaction between SDB and age was observed in a basic model with regards to stroke outcomes (*P* for interaction > 0.2). The association between SDB and stroke outcomes did not differ by sex (all *P* for interaction > 0.2), and all analyses were conducted for men and women combined. The proportion of missing data on the potential confounders used in the main analysis was less than 3% except for smoking status which had less than 7% of missing values. A separate category was created for each variable containing missing values. Potential confounders were selected using directed acyclic graphs [26] based on our a priori knowledge of the relationships among potential confounders, intermediate variables, exposure, and outcome variables, and on existing information regarding factors associated with stroke and SDB [1, 25]. All statistical tests were two sided and p values below 0.05 were considered statistically significant. All statistical analyses were performed using Stata version 15.1 (StataCorp, College Station, TX, USA).

The MR analysis was conducted using the random-effects inverse-variance weighted (IVW) method and supplemented with the weighted median, MR-Egger and MR Pleiotropy Residual Sum and Outlier (PRESSO) methods. The MR-Egger method was used to assess directional pleiotropy [7], whereas the MR-PRESSO method was used to detect potential outlier SNPs [27]. Reported odds ratios (OR) with corresponding 95% confidence intervals (CI) were scaled to one unit increase in log odds of genetic predisposition to sleep apnea. The MR analysis was performed using the mrrobust package in Stata (StataCorp LP, College Station, TX) [24] and the TwoSampleMR and MR-PRESSO packages in R (R Foundation for Statistical Computing, Vienna, Austria) [31].

## Results

### Cohort study

The cohort consisted of 23,050 men and 18,692 women. Among them, 3259 participants (7.8%) reported cessation of breathing and 9569 (22.9%) indicated disturbing snoring. 30.7% of men and 16% of women reported having at least one SDB characteristic (snoring or cessation of breathing); 9.2% of men and 3.6% of women indicated that they experienced both snoring and cessation of breathing. Baseline characteristics of study participants according to SDB are shown in Table 1. Compared with those without snoring or cessation of breathing, individuals who reported these SDB symptoms were somewhat younger, were more likely to be men, had higher alcohol intake, were more likely to be former or current cigarette smokers, were less physically active, had higher BMI, and were more likely to have history of hypertension, hypercholesterolemia, and diabetes.

**Table 1:** Baseline characteristics of the study population according to the presence of sleep-disordered breathing (SDB) symptoms

Characteristics	Presence of SDB symptoms*	
	No	Yes
Number of participants	31,712	10,030
Age at baseline, years, mean (SD)	70.1 (8.0)	67.5 (7.0)
Men, %	50.4	70.5
Education > 12 years, %	22.1	21.7
Cigarette smoking, %		
Former smokers	34.8	43.9
Current smokers	8.1	10.2
Alcohol intake ≥ 15 drinks/week, %	3.0	5.2
Walking/bicycling > 40 min/day, %	35.5	29.8
Exercise ≥ 2 h/week, %	16.0	14.4
Body mass index, kg/m^2^, %		
25.0–29.9	40.0	47.4
≥ 30.0	11.1	19.0
Hypertension, %	39.6	43.0
Hypercholesterolemia, %	23.4	29.0
Diabetes, %	8.5	9.8

*SD* standard deviation

*Participants reported that at least one SDB symptom (cessation of breathing or disturbing snoring) occurred often, mostly or always

The number of incident stroke events during up to 8 years of follow-up is shown in Tables 2 and 3. In the multivariable model, having at least one SDB symptom was associated with increased risks of total stroke and of ICH, but not with ischemic stroke or SAH (Table 2). The presence of both SDB symptoms was associated with increased risk of total stroke in the model adjusted for age, sex and BMI (Table 3). However, this association was only marginally statistically significant after adjustment for all potential confounders (Table 3). The analysis of the relationship of number of SDB-related symptoms and risk of stroke, confirmed that having either snoring or cessation of breathing, was linked to higher risk of ICH. However, having both SDB-related symptoms was not associated with risk of ICH (*p* > 0.05), although the direction of the association was the same (Table 3). The association between number of SDB symptoms and SAH is not presented in Table 3 due to small number of cases in exposure groups. Sensitivity analysis, with additional adjustment for cohabitation status (no/yes), revealed similar results with no change in the HR or a slight change in the second decimal point (data not shown).

**Table 2:** Hazard ratios (95% confidence intervals) of stroke according presence of sleep-disordered breathing (SDB) in the entire study population, follow-up 2009–2017

Outcome and model	At least one SDB symptom^a^	
	No	Yes
Total stroke^b^		
Total number of cases	1758	554
Age and sex-adjusted model	1.00 (reference)	**1.17 (1.06–1.29)**
Multivariable model^c^	1.00 (reference)	**1.14 (1.03–1.26)**
Multivariable model 2^d^	1.00 (reference)	**1.12 (1.02–1.24)**
Total ischemic stroke		
Total number of cases	1483	443
Age and sex-adjusted model	1.00 (reference)	**1.12 (1.00–1.24)**
Multivariable model^c^	1.00 (reference)	1.08 (0.97–1.21)
Multivariable model 2^d^	1.00 (reference)	1.06 (0.95–1.19)
Intracerebral hemorrhage (ICH)		
Total number of cases	200	97
Age and sex-adjusted model	1.00 (reference)	**1.63 (1.27–2.09)**
Multivariable model^c^	1.00 (reference)	**1.59 (1.23–2.05)**
Multivariable model 2^d^	1.00 (reference)	**1.59 (1.23–2.05)**
Subarachnoid hemorrhage (SAH)		
Total number of cases	57	13
Age and sex-adjusted model	1.00 (reference)	0.86 (0.46–1.59)
Multivariable model^c^	1.00 (reference)	0.83 (0.44–1.53)
Multivariable model 2^d^	1.00 (reference)	0.79 (0.42–1.47)

Bold values indicate* P* < 0.05

*CI* confidence interval; *HR* hazard ratio

^a^Participants reported that at least one SDB symptom (cessation of breathing or disturbing snoring) occurred often, mostly, or always

^b^Includes ischemic stroke, intracerebral hemorrhage, subarachnoid hemorrhage, and undefined type of stroke

^c^The Cox proportional hazards regression model was adjusted for age (underlying time scale), sex (as a stratification variable), and body mass index

^d^The Cox proportional hazards regression model was adjusted for age (underlying time scale), sex (as a stratification variable), body mass index, education, smoking status, alcohol consumption, walking/bicycling, exercise, and history of hypertension, hypercholesterolemia, and diabetes

**Table 3:** Hazard ratios (95% confidence intervals) of stroke according to number of sleep-disordered breathing (SDB) symptoms, follow-up 2009–2017

Outcome and model	Number of SDB symptoms^a^		
	No	Snoring or cessation of breathing	Both snoring and cessation of breathing
Total stroke^b^			
Total number of cases	1758	395	159
Age and sex-adjusted model	1.00 (reference)	**1.14 (1.02–1.28)**	**1.25 (1.06–1.47)**
Multivariable model^c^	1.00 (reference)	1.12 (1.00–1.25)	**1.20 (1.02–1.42)**
Multivariable model 2^d^	1.00 (reference)	1.10 (0.99–1.24)	1.17 (0.99–1.38)
Total ischemic stroke			
Total number of cases	1483	315	128
Age and sex-adjusted model	1.00 (reference)	1.09 (0.96–1.23)	1.20 (1.00–1.44)
Multivariable model^c^	1.00 (reference)	1.06 (0.94–1.20)	1.15 (0.96–1.38)
Multivariable model 2^d^	1.00 (reference)	1.04 (0.92–1.18)	1.12 (0.93–1.34)
Intracerebral hemorrhage (ICH)			
Total number of cases	200	72	25
Age and sex-adjusted model	1.00 (reference)	**1.67 (1.27–2.20)**	1.51 (0.99–2.31)
Multivariable model^c^	1.00 (reference)	**1.65 (1.25–2.17)**	1.44 (0.94–2.21)
Multivariable model 2^d^	1.00 (reference)	**1.66 (1.25–2.19)**	1.42 (0.93–2.19)

Bold values indicate* P* < 0.05

*CI* confidence interval; *HR* hazard ratio

^a^Cessation of breathing and disturbing snoring were reported to occur often, mostly or always

^b^Includes ischemic stroke, intracerebral hemorrhage, subarachnoid hemorrhage, and undefined type of stroke

^c^Adjusted for age (underlying time scale), sex (as a stratification variable), and body mass index

^d^Adjusted for age (as the underlying time scale), sex (as a stratification variable), body mass index, education, smoking status, alcohol consumption, walking/bicycling, exercise, and history of hypertension, hypercholesterolemia, and diabetes

### Two-sample MR analysis

**Fig. 1:**
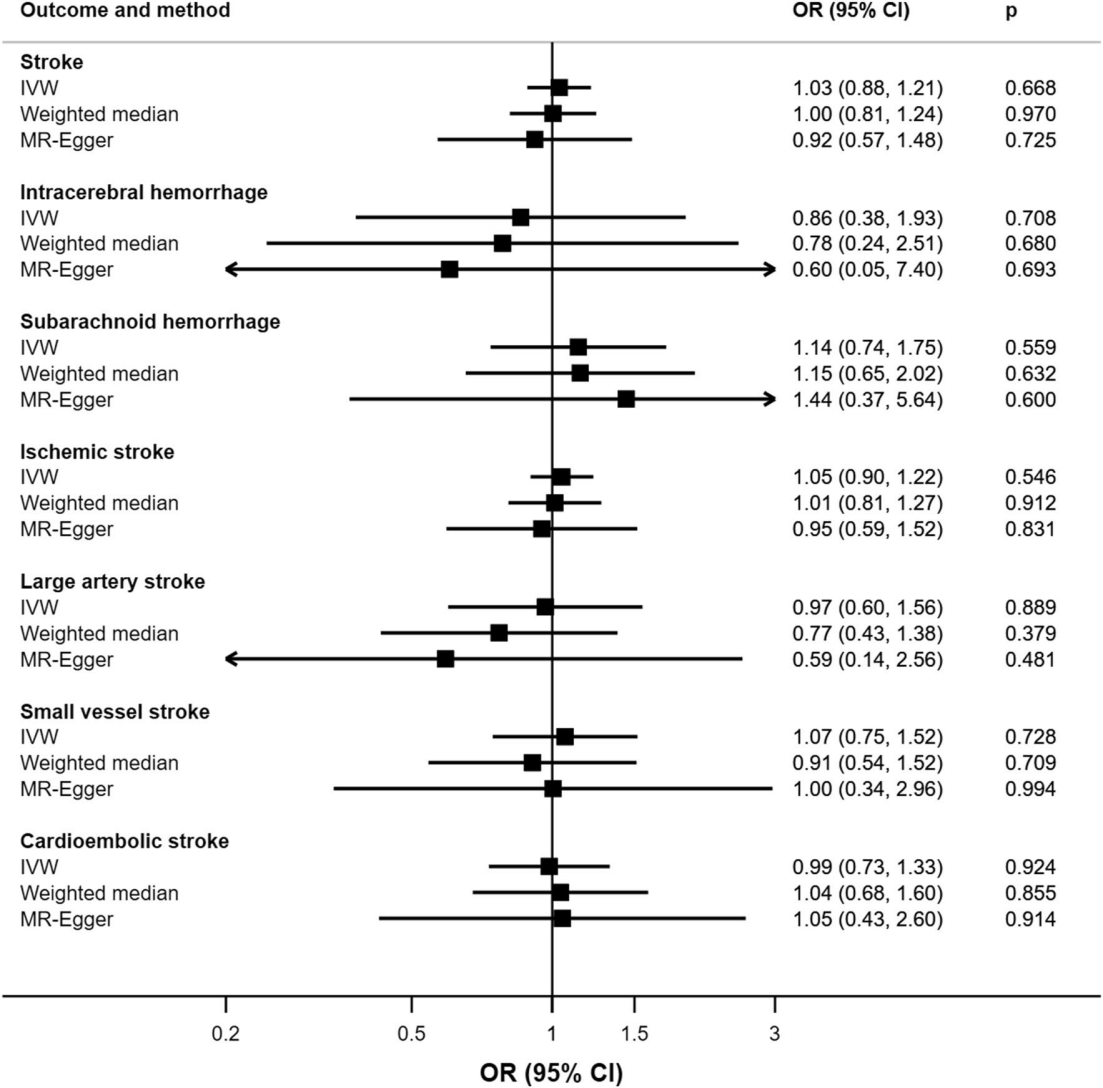
Associations of genetic liability to sleep apnea with overall stroke, ischemic stroke and its subtypes. Odds ratios (OR) are per one unit increase in log odds of sleep apnea. IVW indicates inverse-variance weighted method; and OR, odds ratio

There was no association of genetic liability to sleep apnea with overall risk of stroke or any ischemic or hemorrhagic stroke subtypes (Fig. 1). The lack of association remained in sensitivity analyses. There was no evidence of directional pleiotropy, and no outliers were identified (*Supplementary Table II*).

## Discussion

The present study used a cohort study design and MR approach to evaluate the association between symptoms of SDB and stroke types and subtypes. In the cohort study, an increased risk of total stroke and ICH in particular was observed among individuals with at least one SDB-related symptom (disturbing snoring or cessation of breathing). However, genetic liability to sleep apnea was not associated with overall stroke or ICH in the MR-analyses. There was little evidence of associations of SDB-related traits with risk of ischemic stroke and subarachnoid hemorrhage.

### Comparisons with other studies

Our findings of a positive association between self-reported symptoms of SDB and risk of total stroke are consistent with previous research based on objectively measured OSA [16]. Results of a recent meta-analysis of cohort studies demonstrated that severe OSA was linked to a 2.2-fold higher risk of stroke [30]. However, no association of mild or moderate OSA and risk of stroke was found [30]. Little is known about the link between SDB and risk of specific stroke types. In our study, we did not observe a significant association between symptoms of SDB and risk of ischemic stroke. In contrast, a 6-year longitudinal study of 394 elderly participants did find an association with severe OSA (defined as apnea–hypopnea index, AHI ≥ 30) [18]. The discrepancy in results might be related to differences in the definition of SDB, since we could not properly account for the severity of the SDB. In contrast to our study, a recent retrospective analysis based on clinical data of 10,621 patients with different sleep disorders found that a diagnosis of OSA was associated with a 6.8-fold higher risk of SAH compared to patients with other sleep disorders [33]. Of note, no adjustment for any confounding factor (e.g., sex, age, smoking, BMI) or comparison with a healthy control group was possible in that study [33]. Furthermore, the incidence of SAH in our study was low and we cannot exclude that weak associations might be overlooked. We are not aware of any previous cohort study on SDB in relation to risk of hemorrhagic stroke (ICH or SAH). In the present cohort, those who suffered either from disturbing snoring or cessation of breathing had a 59% higher risk of ICH compared to individuals without SDB-related symptoms. The lack of association between having both disturbing snoring and cessation of breathing and the risk of ICH, may be due to small number of cases in this subgroup (*n* = 25).

Our secondary MR analyses showed no significant association between genetic liability to sleep apnea and stroke. However, these findings in relation to ICH should be interpreted with caution due to the small sample size and low precision. Further cohort and MR studies based on larger GWAS are needed to investigate the relationship between SDB and the risk of hemorrhagic stroke.

### Potential mechanisms

The mechanisms underlying the link between SDB and stroke may involve several pathways. SDB is associated with sleep fragmentation, intermittent hypoxia, inflammation, oxidative stress, endothelial dysfunction [21, 28] and decrease in the nocturnal dipping of blood pressure, [9] all of which may contribute to increased risk of stroke. ICH is usually caused by rupture of blood vessels that have degenerated, possibly due to long-term hypertension [2]. In addition, a recent meta-analysis of 28 case–control studies, indicated that OSA was associated with a decreased flow-mediated dilatation, a measure of endothelial dysfunction [28], which may play a role in brain changes related to cerebral small vessel disease [20]. Such changes may predispose to the poorer functional outcomes following ICH [13].

## Strength and limitations

Important strengths of our cohort study are the large sample size and large number of incident stroke cases objectively assessed through linkage to nationwide population-based registers; complete case identification and no loss to follow-up; and the ability to adjust for important confounders. In addition, the association of symptoms of SDB with several stroke subtypes was investigated, and both men and women were included in this study. Several limitations, however, apply to the present observational study. The information on subtypes of ischemic stroke was not available and the incidence of SAH and ICH among individuals with symptoms of SDB was low, which could increase the risk of type 2 error. SDB-related symptoms were determined based on self-reports and we were unable to determine the severity of the SDB. This could explain the smaller magnitude of associations between SDB symptoms and, for example, total stroke compared to other studies utilizing objective measures (e.g., polysomnography).

Self-reported snoring and cessation of breathing were used here as a proxy for SDB. Already in 1991 it was shown that, independent of age and sex, self-reported snoring frequency is associated with a higher likelihood of sleep apnea as assessed by polysomnography [5]. Hence, snoring is included in all screening instruments for OSA and widely used in epidemiological studies when the objective evaluation with polysomnography is not possible, e.g., due to large sample size. When creating a scoring scale for SDB, the questions on snoring and breathing cessation turned out to have the highest correlation with a scoring scale with adequate reliability for sleep-disordered breathing as identified with polysomnography [11]. In a recent study, the predictive value of several OSA screening instruments was explored, and the authors concluded that self-reported observed apnea had the strongest association with moderate sleep apnea (apnea–hypopnea index ≥ 15) [14]. However, although both snoring and cessation of breathing have high predictive value for OSA, the exact validity remains unknown; and as no polysomnography was performed in this cohort we have no information on severity of SDB.

Another limitation is that we have no information on treatment for SDB during the follow-up period, and the association between SDB symptoms and stroke could thereby be underestimated in our study. Finally, in view of the observational nature of this study, we cannot rule out residual and unmeasured confounding (e.g., additional potential confounders, such as medication, were not included in the analyses).

The major strength of our MR study includes the large number of overall stroke and total ischemic stroke cases and data on etiologic subtypes of ischemic stroke; and that potential confounding and reverse causality, which can bias the findings from conventional observational studies, were reduced by the use of genetic variants as proxy measures for sleep apnea. In addition, our MR analysis was restricted to European descent individuals which reduced potential bias due to population stratification. Finally, methods to correct for possible pleiotropy and identify potential outliers SNPs were used. An important limitation is that the analysis of the association between genetically predicted sleep apnea and ICH was based on a small sample, and we cannot exclude that a weak association was overlooked. Thus, further large studies in other populations are needed.

## Conclusions

Results from this large-scale cohort study of middle-aged and older individuals showed that self-reported markers of SDB such as snoring and breathing cessations were associated with an increased risk of total stroke and ICH, but not with ischemic stroke or subarachnoid hemorrhage. Two-sample MR analysis showed no evidence of an association of sleep apnea and any stroke outcome but had limited power in the analysis of ICH. Thus, the association between SDB and ICH needs further study.

## Supplementary Information

Below is the link to the electronic supplementary material.

Supplementary file1 (PDF 138 KB)

## Data Availability

The data that support findings of the prospective cohort study are available upon application to the Swedish Infrastructure for Medical Population-Based Life-Course Environmental Research (https://www.simpler4health.se). Data used for the MR analyses are publicly available from the MEGASTROKE consortium (https://www.megastroke.org/) [15], the International Stroke Genetics Consortium (https://strokegenetics.org/) [29] and a meta-analysis of Genome-Wide Association Studies [4].
