# Supplementary material for: Sleep-disordered breathing-related symptoms and risk of stroke: cohort study and Mendelian randomization analysis
Source: J Neurol. 2021 Oct 1;269(5):2460–8. doi: 10.1007/s00415-021-10824-y (PMC9021054; doi:10.1007/s00415-021-10824-y)
Supplement: Supplementary file 1 — Supplementary file1 (PDF 138 KB) [file 415_2021_10824_MOESM1_ESM.pdf]

SUPPLEMENTAL MATERIAL

Sleep-Disordered Breathing-Related Symptoms and Risk of Stroke: Cohort Study and Mendelian Randomization Analysis

Olga E Titova, Shuai Yuan, John A Baron, Eva Lindberg, Karl Michaëlsson, Susanna C Larsson

Supplemental Table I. Details of the single nucleotide polymorphisms used as instrumental variables in the Mendelian randomization analysis

Supplemental Table II. Association between genetically predicted sleep apnea and stroke in the primary inverse-variance weighted analysis and in sensitivity analyses using other MR methods

Supplemental Table I. Details of the single nucleotide polymorphisms used as instrumental variables in the Mendelian randomization analysis

rsID	Nearest gene(s)	Chr	Position	EA	OA	Beta	SE
rs543874	FAM5B, SEC16B	1	177889480	G	A	-0.027	0.005
rs11205802	MACF1	1	39699114	T	C	-0.02	0.004
rs72904209	NR4A2	2	157046432	T	C	-0.036	0.006
rs13021737	FAM150B, TMEM18	2	632348	G	A	-0.022	0.005
rs1554654	ABHD5, TOPAZ1	3	44044344	T	C	-0.018	0.004
rs1403848	ROBO2	3	77609655	C	A	0.016	0.004
rs7715167	TLX3, NPM1	5	170778824	T	C	0.024	0.004
rs10075809	CEP120	5	122703026	C	A	0.016	0.004
rs79932406	XKR9	8	71607667	T	G	0.016	0.004
rs7005777	PEX2	8	78233600	T	G	0.018	0.004
rs8176749	ABO	9	136131188	T	C	-0.032	0.007
rs12683343	HSPA5, GAPVD1	9	128012939	G	A	0.015	0.004
rs1444789	GATA3	10	9064361	T	C	-0.037	0.005
rs7107532	METTL15	11	28480924	G	A	-0.027	0.004
rs12805133	SPTBN2	11	66483265	G	A	0.019	0.004
rs6265	BDNF	11	27679916	T	C	-0.022	0.005
rs11821161	GRM5, TYR	11	88888262	T	C	-0.015	0.004
rs9783497	MSRB3	12	65830349	G	A	-0.04	0.004
rs2958153	PTGES3	12	57081517	G	A	-0.031	0.004
rs11176018	AC090673.2,LLPH	12	66451720	T	G	0.018	0.004
rs7138383	C12orf42	12	103724090	G	A	0.017	0.004

rs592333	DLEU7	13	51340315	G	A	0.04	0.004
rs11634019	ISL2	15	76634680	T	C	0.027	0.004
rs1436047	RP11-457D20.2	16	60618247	G	A	-0.025	0.004
rs1136070	HN1L	16	1751935	T	C	0.037	0.007
rs11075985	FTO	16	53805207	C	A	-0.017	0.004
rs879620	ADCY9	16	4015729	T	C	-0.016	0.004
rs227731	NOG, C17orf67	17	54773238	T	G	-0.026	0.004
rs12603115	SKAP1	17	46248994	T	C	-0.018	0.004
rs4987719	BCL2	18	60960310	T	C	0.061	0.011
rs35445111	TSHZ3,ZNF507	19	32172047	G	A	-0.048	0.007
rs6113592	PAX1,FOXA2	20	22229505	G	A	-0.02	0.004
rs17794954	ZFP64,TSHZ2	20	50966307	T	C	-0.022	0.005
rs6038517	FERMT1,BMP2	20	6458205	G	A	0.017	0.005
rs2735309	HMGN1	21	40715313	T	C	-0.021	0.004

Chr, chromosome; EA, effect allele; OA, other allele; SE, standard error.

Supplemental Table II. Sensitivity analyses, MR-Egger and MR Pleiotropy Residual Sum and Outlier (PRESSO) methods

		MR-Egger intercept			MR-PRESSO					
**Stroke type or subtype**	**No. of SNPs**	**Intercept**	**SE**	**p**	**Outliers**	**Beta**	**SE**	**p**	**P global test**	**P distortion test**
Intracerebral hemorrhage	33	0.009	0.031	0.772	NA	NA	NA	NA	0.479	NA
Subarachnoid hemorrhage	33	-0.006	0.017	0.721	NA	NA	NA	NA	0.299	NA
Stroke*	35	0.003	0.006	0.602	NA	NA	NA	NA	0.194	NA
Ischemic stroke	35	0.003	0.006	0.663	NA	NA	NA	NA	0.496	NA
Large artery stroke	35	0.013	0.018	0.485	NA	NA	NA	NA	0.024	NA
Small vessel stroke	35	0.002	0.013	0.910	NA	NA	NA	NA	0.667	NA
Cardioembolic stroke	35	-0.002	0.011	0.882	NA	NA	NA	NA	0.720	NA

*Ischemic stroke and intracerebral hemorrhage

MR, Mendelian randomization; SNP, single-nucleotide polymorphism; SE, standard error
